# TDP-43 Is Elevated in Plasma Neuronal-Derived Exosomes of Patients With Alzheimer’s Disease

**DOI:** 10.3389/fnagi.2020.00166

**Published:** 2020-06-04

**Authors:** Nan Zhang, Dongmei Gu, Meng Meng, Marc L. Gordon

**Affiliations:** ^1^Department of Neurology, Tianjin Medical University General Hospital, Tianjin Neurological Institute, Tianjin, China; ^2^Department of Neurology, Tianjin Medical University General Hospital Airport Hospital, Tianjin, China; ^3^Department of Clinical Laboratory Medicine, Tianjin Medical University General Hospital, Tianjin, China; ^4^The Litwin-Zucker Research Center, The Feinstein Institutes for Medical Research, Northwell Health, Manhasset, NY, United States; ^5^Donald and Barbara Zucker School of Medicine at Hofstra/Northwell, Hempstead, NY, United States

**Keywords:** TDP-43, Alzheimer’s disease, exosome, cognitive function, neuropsychiatric symptoms, APOE

## Abstract

**Background:**

Recently, TDP-43 has been recognized as a common proteinopathy in the “oldest old” and a neuropathological comorbidity in patients with Alzheimer’s disease (AD). However, since it has a low concentration in cerebrospinal fluid, the presence of TDP-43 in AD is rarely investigated *in vivo*.

**Methods:**

Twenty-four patients with amyloid PET confirmed AD and 15 healthy controls (HCs) were included in this study. TDP-43 level in plasma neuronal-derived exosomes (NDEs) was measured by enzyme-linked immunosorbent assay.

**Results:**

TDP-43 level was elevated in patients with AD compared with HCs (median 1.08 ng/ml, IQR 0.72–1.37 ng/ml vs. median 0.66 ng/ml, IQR 0.48–0.76 ng/ml, *P* = 0.002). There was no correlation between TDP-43 level and cognitive function, neuropsychiatric symptoms or APOE genotype in patients with AD.

**Conclusion:**

This study demonstrated increased TDP-43 accumulation in AD patients by examining plasma NDEs, which may provide a window into the effects of TDP-43 on AD progression.

## Introduction

TAR DNA binding protein of 43 kDa (TDP-43), which plays a fundamental role in exon skipping, nuclear transcription, splicing and stability of RNA transcripts, micro-RNA processing, and other cellular functions (Buratti et al., 2004; Buratti and Baralle, 2008; Buratti et al., 2010) has been identified in an abnormal phosphorylated state in cellular inclusions and is associated with neurodegeneration and cognitive impairment in the majority of patients with tau-negative frontotemporal lobar degeneration (FTLD) and nearly all patients with amyotrophic lateral sclerosis (ALS) (Neumann et al., 2006; Feneberg et al., 2018). Moreover, TDP-43 is considered to be an independently pathogenic proteinopathy causing an amnestic dementia syndrome, which was recently named limbic-predominant age-related TDP-43 encephalopathy (LATE) (Nelson et al., 2019).

In the past decade, several studies reported that TDP-43 accumulates pathologically in the brains of patients with AD. The proportion of AD patients with TDP-43 pathology has been reported to range from 19 to 73.9% (Josephs et al., 2014a; McAleese et al., 2017). The pathological comorbidity of TDP-43 is first observed in the medial temporal lobe and eventually spreads to occipitotemporal cortex, basal ganglia, and frontal neocortex in patients with AD (Bayram et al., 2019). In a large autopsy sample, a pathological diagnosis of AD mixed with TDP-43 was the most common mixed pathology in subjects with AD (compared to pathological AD mixed with infarcts, arteriolosclerosis, Lewy bodies or hippocampal sclerosis) (James et al., 2016).

The burden of TDP-43 accumulation was observed to be correlated with progression of AD, such as volumetric reductions in the hippocampal and entorhinal cortex, decline in memory, naming, general cognition, and global function, particularly during the early phases of neurodegeneration (Josephs et al., 2014a, b, 2015, 2016, 2017; McAleese et al., 2017; Flanagan et al., 2018). Furthermore, previous pathological studies have provided conflicting evidence as to whether TDP-43 might be a risk factor or a protective factor in terms of neuropsychiatric symptoms in patients with AD (Vatsavayi et al., 2014; Sennik et al., 2017; Bayram et al., 2019).

Although the plasma concentration of TDP-43 has been observed to be elevated in patients with FTLD, since TDP-43 has a low concentration in cerebrospinal fluid (CSF) and may mainly originate from blood, its correlation with neurodegeneration in AD is still controversial (Foulds et al., 2008; Foulds et al., 2009; Williams et al., 2017). Exosomes are a subtype of extracellular vesicles that arise from a wide range of cells and contain molecular cargo, including a variety of proteins (Shah et al., 2018). It has been demonstrated that TDP-43 is secreted via exosomes in neuronal cells contributing to both propagation and clearance of TDP-43 in ALS brains (Iguchi et al., 2016) and can be detected in exosomes from CSF, which originate in the brain (Feneberg et al., 2014).

Neuronal-derived exosomes (NDEs) have been successfully isolated from plasma, and analyzed for the expression of AD biomarkers, such as Aβ, tau, cellular survival factors, lysosomal proteins, insulin receptor substrate and synaptic proteins in previous studies (Fiandaca et al., 2015; Goetzl et al., 2015a, b, 2016; Kapogiannis et al., 2015; Mustapic et al., 2017). In the present study, we aimed to measure expression of TDP-43 in patients with AD by isolating and analyzing plasma NDEs, and to further explore the association between TDP-43 and cognitive function, neuropsychiatric symptoms and APOE genotype.

## Materials and Methods

### Participants

Twenty-four patients with AD and 15 healthy controls (HCs) recruited from our longitudinal MRI study of AD and subcortical ischemic vascular dementia were included in this study. Patients with AD met the International Working Group-2 (IWG-2) diagnostic criteria for AD (Dubois et al., 2014), had an amnestic symptom and an amyloid-positive ^11^C-Pittsburgh compound B (PiB) PET scan, were aged 50–85 years, with a Mini-Mental State Examination (MMSE) score of 10–26 and a Clinical Dementia Rating (CDR) score of 0.5–2. Patients whose cognitive decline was caused by other neurological diseases, mental disorders, or medical conditions, such as FTLD, dementia with Lewy bodies, Parkinson’s disease, vascular dementia, multiple sclerosis, severe depression, vitamin B12 deficiency, or thyroid dysfunction, were excluded. Age- and sex-matched HCs had no complaint of cognitive decline, with an MMSE score >24 and a CDR score = 0. All participants underwent comprehensive neuropsychological testing and multimodal brain MRI scans, and blood samples were collected for further investigation. This study was approved by the Ethics Committee of Tianjin Medical University General Hospital. Written informed consent was obtained from all participants.

### Neuropsychological Assessment

A neuropsychological battery was performed to test various cognitive domains for all subjects, including the Rey Auditory Verbal Learning Test (AVLT) (Wu et al., 2009), the Symbol Digit Modalities Test (SDMT) (Smith, 1982), the Trail Making Test-A (TMT-A) and B (TMT-B) (Rossini and Karl, 1994), the Stroop test (Stroop, 1935), the Animal Verbal Fluency Test (AFT) (Mok et al., 2004), the Controlled Oral Word Association Test (COWAT) (Zhang et al., 2015), the Boston Naming Test (BNT) (Williams et al., 1989) and the Benton Judgment of Line Orientation (JLO) (Benton et al., 1994). All of the above tests have a higher score indicating better performance on the specific tasks, except for the TMT-A and TMT-B with an opposite implication. Raw scores were converted to Z scores using the mean and SD of the HC group. Five main cognitive domains were calculated: (1) memory composite = average Z score of total learning, delayed recall and recognition on the AVLT; (2) language composite = average of the AFT, the COWAT and the BNT; (3) attention and information processing speed composite = average of the SDMT and the TMT-A; (4) executive function composite = average of the TMT-B and the Stroop color-word test; (5) visuospatial function = Z score of the JLO.

Behavioral and psychological symptoms of dementia (BPSD) were assessed using the 12-item Neuropsychiatric Inventory (NPI) (Cummings et al., 1994). Apart from the total score of the NPI, the presence of motor disturbance and four symptom clusters (Sayegh and Knight, 2014) including psychosis (delusions, hallucinations), hyperactivity (agitation, disinhibition, irritability), affect (depression, anxiety), and apathy/vegetative (apathy, sleep, appetite), was also analyzed in this study.

### APOE Genotyping

A 500 μl sample of blood was collected in ethylenedi- aminetetraacetic acid-containing (EDTA) vacutainer tubes from all participants. Genomic DNA was extracted using the TIANamp Blood DNA Kit (Tiangen Biotech Co., Ltd., Beijing, China) following the manufacturer’s protocol. Then polymerase chain reaction amplification of the APOE gene was followed by using genomic DNA. The accuracy of genotyping was further confirmed with Sanger sequencing by using an ABI 3730xl DNA analyzer (Applied Biosystems) in Allwegene Clinical Testing Laboratory (Tianjin, China). The sequences were analyzed using the software Sequencing Analysis 5.2. Primer sequences are listed in Supplementary Table 1. The APOE status of all participants was determined by two single nucleotide polymorphisms (rs429358 and rs7412) that define the epsilon 2, 3, and 4 alleles.

### Exosome Isolation and Identification

The isolation of NDEs was performed according to the methods previously developed by Goetzl (Fiandaca et al., 2015). 0.5 ml of plasma was incubated with 0.15 ml of thromboplastin-D (Fisher Scientific, Inc., Hanover Park, IL, United States) at room temperature for 60 min, followed by the addition of 0.35 ml of calcium- and magnesium-free Dulbecco’s balanced salt solution (DBS^–2^) with 20 μl protease inhibitor cocktail (Roche Applied Sciences, Inc., Indianapolis, IN) and 5 μl phosphatase inhibitor cocktail (Pierce Halt, Thermo Scientific, Inc., Rockford, IL, United States). After centrifugation at 3000 × g for 20 min, supernates were mixed with 252 μl of ExoQuick exosome precipitation solution (EXOQ; System Biosciences, Inc., Mountainview, CA, United States), and incubated for 1 h at 4°C. Resultant exosome suspensions were centrifuged at 1500 × g for 30 min at 4°C and each pellet was resuspended in 350 μl of DBS^–2^ with inhibitor cocktails.

Each sample was mixed with 50 μl of 3% bovine serum albumin (BSA) (Thermo Scientific, Inc.) and was incubated for 1 h at 4°C with 1μg of mouse anti-human CD171 antibody (L1CAM neural adhesion protein, eBio5G3, Biotin, eBioscience, San Diego, CA, United States), then followed by addition of 25 μl streptavidin-agarose resin (Thermo Scientific, Inc.) plus 50 μl of 3% BSA for 30 min at 4°C. After centrifugation at 400 × g for 10 min at 4°C and removal of the supernates, each pellet was suspended in 50 μl of 0.05 M glycine-HCl (pH 3.0) by vortex-mixing for 10 s. Each suspension was then combined with 0.5 ml M-PER mammalian protein extraction reagent that had been adjusted to pH 8.0 with 1 M Tris-HCl (pH 8.6) and the inhibitor cocktails followed by incubation for 10 min at 37°C with vortex-mixing for 15 s and was stored at −80°C before enzyme-linked immunosorbent assay (ELISA).

Neuronal-derived exosomes were identified by both transmission electron microscopy and a nanoparticle tracking system. Transmission electron microscopy measurement was conducted using a Talos F200c electron microscope (FEI, United States) at an acceleration voltage of 200 kV to characterize the size and shape of NDEs. In addition, exosomes were visualized with a NanoSight 500 instrument (NanoSight, Amesbury, United Kingdom) and characterized according to the size distribution of vesicles.

### TDP-43 Assay

TDP-43 protein level in plasma NDEs was assayed by ELISA kits (Signalway Antibody, College Park, MD, United States). Human CD81 (Cusabio-American Research Products, Inc.) was used for normalization of TDP-43 concentration. The mean value of all CD81 levels was set at 1.00, and the relative value for each sample was used to normalize their recovery.

### Statistical Analysis

Statistical analyses were performed using SPSS 13.0 (SPSS Inc., United States). Demographic and clinical data of patients with AD and HCs were analyzed using Pearson chi-square test for categorical variables or independent-sample t-test for continuous variables. The difference in CD81 and TDP-43 level between the two groups was analyzed using Mann-Whitney U-tests. Partial correlation analyses were conducted to test the correlations between TDP-43 and the neuropsychological scores in patients with AD, controlling for age, sex and educational level. With respect to the NPI clusters and APOE analyses, dichotomized classification was used according to the presence or absence of the symptoms, or the epsilon 4 allele carrier status. Then, Mann-Whitney U-tests were used to test the difference in TDP-43 level between AD patients with and without the specific neuropsychiatric symptoms, and between APOE epsilon 4 carriers vs. non-carriers within the AD group. All the tests were two-tailed, and values of *P* < 0.05 were regarded as significant.

## Results

### Demographic and Clinical Features

Twenty-four patients with AD (age range: 53–84, mean age: 67.8 ± 8.2 years, median age: 68 years, 17 females) and 15 HCs (age range: 55–77, mean age: 64.8 ± 6.0 years, median age: 64 years, 10 females) were included in this study. There was no significant difference in age, sex or education between the AD patients and HCs. The AD group had a significantly lower score on MMSE (16.3 ± 6.1 vs. 27.7 ± 1.7) and a much higher proportion of APOE ε4 carriers (3 with 4/4, 10 with 3/4, 10 with 3/3, 1 with 2/3 vs. 0 with 4/4, 2 with 3/4, 9 with 3/3, 4 with 2/3) compared with the HC group. Table 1 presents the demographic characteristics of the two groups.

**TABLE 1:** Demographic and clinical features of patients with AD and HCs.

	**AD *N* = 24**	**HC *N* = 15**	***P***
Age, y	67.8 ± 8.2	64.8 ± 6.0	0.228
Sex, F/M	17/7	10/5	0.784
Education, y	11.0 ± 3.6	12.6 ± 2.4	0.125
APOE ε4 carrier/non-carrier	11/13	2/13	0.036
MMSE	16.3 ± 6.1	27.7 ± 1.7	0.000

**Age, education and MMSE are provided as mean ± SD. AD, Alzheimer’s disease; HC, healthy control; MMSE, Mini-Mental State Examination.**

### TDP-43 Level in Exosomes

**FIGURE 1:**
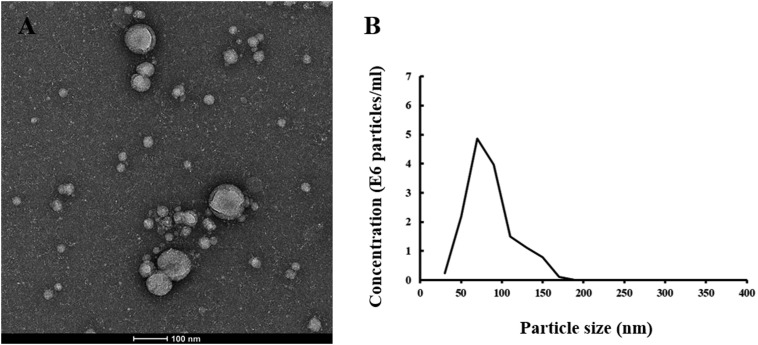
Plasma neuronal-derived exosomes identified with TEM and NTA. **(A)** A representative image detected with transmission electron microscopy of exosomes extracted from an AD patient. The scale bar equals 100 nm. **(B)** A representative plot of size/concentration determined with nanoparticle tracking analysis for plasma exosomes derived from an AD patient.

**FIGURE 2:**
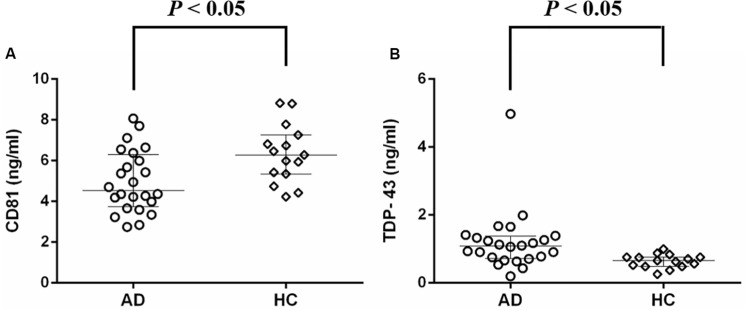
Protein concentrations of plasma neuronal-derived exosomes detected with ELISA. **(A)** Median CD81 level was lower in patients with AD compared to HCs. **(B)** Median normalized TDP-43 concentration was higher in patients with AD compared to HCs. AD, Alzheimer’s disease; HC, healthy control.

Neuronal-derived exosomes were identified with an electron microscope (Figure 1A) and a nanoparticle tracking system (Figure 1B). The size and shape of plasma NDEs from AD patients are similar to those previously reported (Winston et al., 2016). CD81, an exosome membrane marker, was measured and used to normalize the concentration of NDEs for all participants. The AD group showed a lower CD81 level (median 4.53 ng/ml, interquartile range (IQR) 3.74–6.28 ng/ml vs. median 6.28 ng/ml, IQR 5.35–7.26 ng/ml, Mann-Whitney U = 91.0, *P* = 0.01) than the HC group (Figure 2A). Normalized plasma neuronal-derived exosomal concentration of TDP-43 was higher in patients with AD compared to HCs (median 1.08 ng/ml, IQR 0.72–1.37 ng/ml vs. median 0.66 ng/ml, IQR 0.48–0.76 ng/ml, Mann-Whitney *U* = 71.0, *P* = 0.002) (Figure 2B).

### The Association Between TDP-43 and Cognitive Function, Neuropsychiatric Symptoms, and APOE Genotype in Patients With AD

Z scores for all cognitive domains, including memory, language, attention and information processing speed, executive function, and visuospatial function, were prominently decreased in patients with AD compared with HCs. TDP-43 level in NDEs did not correlate with the total MMSE score or the Z score of any cognitive domain, after controlling for age, sex and educational level (Table 2).

**TABLE 2:** Correlation between TDP-43 level and cognitive function in patients with AD.

	**Score**	***r***	***P***
MMSE	16.33 ± 6.14*	0.062	0.791
Memory	−3.38 ± 1.36**	0.304	0.181
Language	−2.29 ± 1.81**	0.067	0.772
Information processing speed	−2.37 ± 1.42**	0.056	0.809
Executive function	−1.79 ± 0.90**	0.161	0.485
Visuospatial function	−3.22 ± 2.14**	0.143	0.537

AD, Alzheimer’s disease; MMSE, Mini-Mental State Examination. * Raw score of the MMSE. ** Z score of the cognitive domains.

**FIGURE 3:**
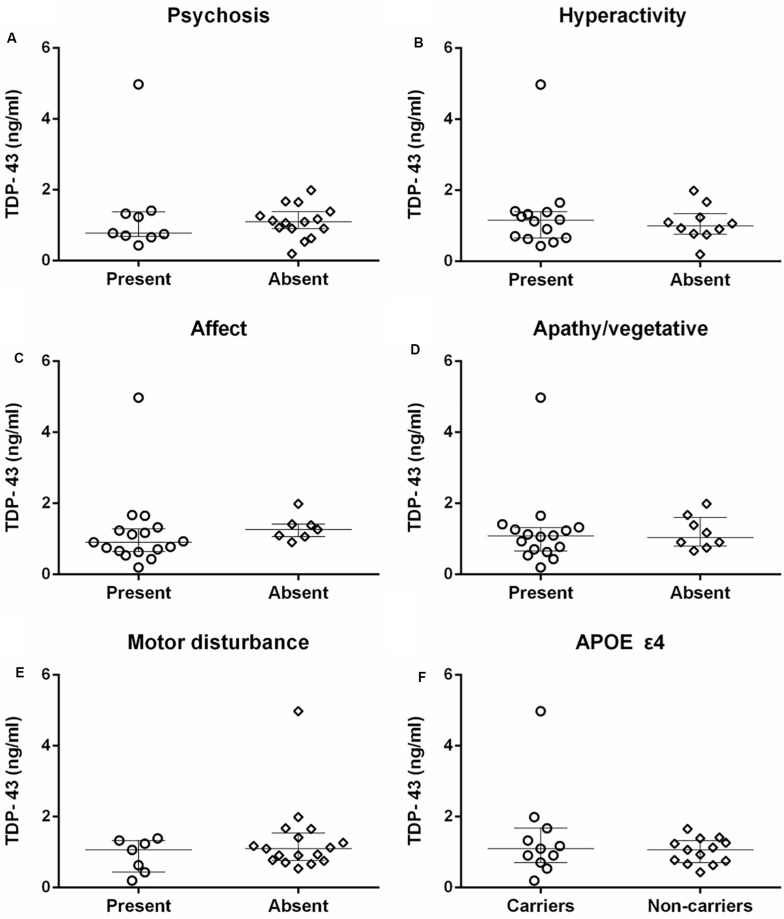
The comparison of TDP-43 concentration between patients with and without specific neuropsychiatric symptom clusters, and APOE ε4 carriers and non-carriers in AD. **(A–E)** There were no correlations between TDP-43 and psychosis, hyperactivity, affect, apathy/vegetative, and motor disturbance measured with NPI in patients with AD (*P* > 0.05). **(F)** The difference in TDP-43 level between APOE ε4 carriers and non-carriers of AD patients was not statistically significant (*P* > 0.05).

There was no significant difference in neuronal-derived exosomal concentration of TDP-43 between AD patients with and without symptoms of any neuropsychiatric cluster according to the NPI, including psychosis (medians 0.78 ng/ml vs. 1.10 ng/ml), hyperactivity (medians 1.15 ng/ml vs. 1.00 ng/ml), affect (medians 0.91 ng/ml vs. 1.26 ng/ml), apathy/vegetative (medians 1.08 ng/ml vs. 1.04 ng/ml) and motor disturbance (medians 1.06 ng/ml vs. 1.10 ng/ml) (Figures 3A–E).

TDP-43 level did not differ between APOE ε4 carriers (median 1.10 ng/ml) and non-carriers (median 1.06 ng/ml) in patients with AD (Figure 3F).

## Discussion

TDP-43 has been increasingly recognized as an independent proteinopathy associated with an amnestic syndrome that can mimic AD, as well as a common neuropathological comorbidity in patients with AD. In the present study, we demonstrated that TDP-43 level in NDEs from plasma is elevated in patients with AD. However, we did not observe any correlation between TDP-43 level and cognitive function, neuropsychiatric symptoms or APOE genotype in AD.

It has been reported that TDP-43 pathology is strongly correlated with advanced AD and arteriosclerotic pathologies in the aged human brain according to a neuropathology data set study (Katsumata et al., 2018). We found that TDP-43 level was increased in patients with AD compared to cognitively healthy individuals through analysis of plasma NDEs, which may reflect neuropathological status. Our present finding supports previous studies that TDP-43 pathology could be a common comorbidity in patients with AD. Recently, it has been observed that there is a correlation between TDP-43 burden and Aβ deposition (Wennberg et al., 2018; Bayram et al., 2019) and increased hippocampal TDP-43 pathology is associated with advanced tau neurofibrillary tangle pathology (Smith et al., 2018) in patients with AD. Moreover, TDP-43 contributed to reducing plaque burden and increasing pre-fibril oligomers of Aβ (LaClair et al., 2016) as well as exacerbating tau aggregation (Davis et al., 2017) in an APP/PS1 mouse background.

TDP-43 has been found to be associated with cognitive decline and dementia conversion in a cohort of older persons without dementia at study entry (Wilson et al., 2013). However, the correlation between TDP-43 and cognitive deficits in patients with AD has not been established. We did not find a relationship between TDP-43 level in plasma NDEs and global cognition measured with the MMSE, or any specific cognitive domain, including memory, language, attention and processing speed, executive function, and visuospatial function. The current National Institute on Aging and Alzheimer’s Association (NIA-AA) research framework posits that the decline in cognitive function of AD is mainly attributable to the interaction of amyloid plaques, neurofibrillary tau deposits and neurodegeneration (Jack et al., 2018) in which TDP-43 is involved but may not be a determinant.

In the present study, TDP-43 level was not associated with any cluster of neuropsychiatric symptoms measured with the NPI, including psychosis, hyperactivity, affect, apathy/vegetative, and motor disturbance. A few previous studies focused on the contribution of TDP-43 to BPSD in patients with AD, but the findings were inconclusive. For instance, TDP-43 was observed to be a risk factor for agitation/aggression assessed by the NPI-Q in AD patients with high pathology load of neurofibrillary tangles (Sennik et al., 2017). In another study using the NPI-Q to evaluate BPSD, TDP-43 was associated with increased severity of aberrant motor behavior and decreased severity of depression, and the correlations between TDP-43 and neuropsychiatric symptoms interacted with amyloid and Lewy body pathologies (Bayram et al., 2019). Additionally, there was no reported correlation between global TDP-43 pathology and psychosis, which was rated with the Consortium to Establish a Registry for Alzheimer’s disease Behavioral Rating Scale. However, TDP-43 in the frontal cortex may have a protective effect regarding the risk of psychosis in patients with AD (Vatsavayi et al., 2014). Therefore, the effect of TDP-43 on BPSD may interact with core AD pathology and other neuropathological changes, and may be dependent on the brain region of its accumulation.

It has been observed that APOE ε4 is associated with TDP-43 burden in community-based individuals (Yang et al., 2018) and patients with AD (Josephs et al., 2014b, 2017) according to post-mortem studies. In another study, although APOE ε4 was directly correlated with TDP-43, this effect was mediated by Aβ and tau (Wennberg et al., 2018). However, we did not identify a relationship between APOE genotype and TDP-43. From our point of view, the effect of APOE genotype on TDP-43 accumulation in patients with AD should be carefully interpreted, since APOE affects core AD neuropathology and cerebrovascular disease, both of which also interact with TDP-43.

In the present study, all participants were recruited from our prospective research study, and all patients with AD had a biomarker-supported diagnosis with PiB PET. However, there are some limitations to our study. L1CAM was used to extract NDEs from plasma, however, it is not exclusively expressed in the CNS, but also in other tissues including skeletal muscle and fat. In addition, the concentration of exosomes identified by CD81 was different between the AD patient group and the HC group, although we standardized TDP-43 level with CD81 for subsequent analysis. This discrepancy could be partly explained by the impact of APOE ε4, which has been observed to reduce exosome expression in mouse and human brain tissues (Peng et al., 2019). In this study, 54.2% of AD subjects were APOE ε4 carriers, compared with only 13.3% of HCs. Moreover, the antibody used in this study recognizes full length TDP-43 protein. We did not assay specific forms of TDP-43, such as phosphorylated or truncated fragments, which should be explored in future investigations. Furthermore, some neuropsychological testing scales have “floor effects” and “ceiling effects.” For instance, several AD patients were unable to complete the TMT-B task within the set time of 300 s; on the other hand, some items were scored 0 for the NPI in very mild patients. These effects may have affected the correlation analyses between TDP-43 and cognitive function and neuropsychiatric symptoms. Finally, the samples size was relatively small, especially for the HCs. Our findings need to be validated in a large population cohort.

## Conclusion

TDP-43 level in plasma NDEs is increased in patients with AD. Although we did not find any correlation between TDP-43 level and cognitive function, neuropsychiatric symptoms or APOE genotype in patients with AD, the relationship between TDP-43 pathology, cognition and BPSD is complicated, and may interact with other neuropathology in the AD context. The measurement of TDP-43 in plasma NDEs is a promising non-invasive biomarker with the potential to provide insight into the role of TDP-43 in neurodegeneration and progression in AD.

## Data Availability Statement

The raw data supporting the conclusions of this article will be made available by the authors, without undue reservation, to any qualified researcher.

## Ethics Statement

The studies involving human participants were reviewed and approved by the Ethics Committee of Tianjin Medical University General Hospital. The patients/participants provided their written informed consent to participate in this study.

## Author Contributions

NZ and DG were responsible for concept, study design, statistical analysis, and drafting of the manuscript. NZ, DG, and MM were responsible for acquisition, analysis, and interpretation of data. NZ and MG substantively revised the manuscript for important intellectual content.

## Conflict of Interest

MG has received research support without direct compensation from MSD (Merck), Eisai, AbbVie, and Janssen, and also served on an advisory board for Eisai. The remaining authors declare that the research was conducted in the absence of any commercial or financial relationships that could be construed as a potential conflict of interest.
