# Supplementary material for: TDP-43 Is Elevated in Plasma Neuronal-Derived Exosomes of Patients With Alzheimer’s Disease
Source: Front Aging Neurosci. 2020 Jun 4;12:166. doi: 10.3389/fnagi.2020.00166 (PMC7287025; doi:10.3389/fnagi.2020.00166)
Supplement: Supplementary file 1 [file Table_1.PDF]

Supplementary Table 1. Primers of APOE gene testing.

Forward Primer	Reverse Primer	Sequencing Primer
TAAGCTTGGCACGGCT GTCCAAGGA	ACAGAATTCGCCCCGG CCTGGTACAC	TAAGCTTGGCACGGCT GTCCAAGGA
